# The United Kingdom Hospital Conference

**Published:** 1906-12-01

**Authors:** 


### THE UNITED KINGDOM HOSPITAL CONFERENCE.

### RESOLUTIONS TO BE PROPOSED.

The hospitals and the joint committees which have been working for two years on the subject of hospital abuse at the instigation of the British Medical Association have organised a conference of consultants and general practitioners, representatives of hospitals and of the chief supporters of the voluntary hospitals, and members of both Houses of Parliament, which will be held on Thursday, December 6, at 2.30 p.m., in the Botanical Theatre, University College, Gower Street. Upwards of 150 representatives have already been appointed to take part in this conference, the aim of which will be to secure a careful and sympathetic consideration of the problem of hospital abuse with a view to the cutting down of the present excessive amount of free medical treatment given by the hospitals, and to place the whole matter upon a basis better calculated to do justice to the interests of the hospitals and their subscribers, the suffering poor, and the members of the medical profession. It is thirty years since Sir Charles Trevelyan read his historic paper on this subject, and it is remarkable that the abuses which Sir Charles stated with such clearness should have met with, practically, no redress, but, on the contrary, that several of them should prevail to an even greater extent to-day than they did thirty years ago. The present conference should lead to adequate reforms, for the reasons that the general practitioners, after four years' steady work, are now in a state of active organisation, and can make their views felt by hospital consultants, whilst the larger subscribers to the hospitals are impressed with the feeling that too much free medical relief exists, and that its volume must be materially cut down, if the hospitals are to continue as voluntary institutions. We believe, that, the managers of the best administered hospitals, at any rate, are fully alive to the evils, and that they too would welcome a modification of system, which, by reducing the numbers admitted to relief, would enable the work to be more efficiently conducted than it can be in present circumstances.

### RESOLUTIONS TO BE SUBMITTED TO THE CONFERENCE.

1. (To be moved by Sir Henry Burdett, K.C.B., and seconded by Dr. Frank M. Pope, F.R.C.P., Chairman of the Hospitals Committee, British Medical Association) That this conference of representatives and supporters of hospitals, medical practitioners, and others interested in hospital administration in Great Britain and Ireland, approves generally of the principles contained in the proposals drawn up by the Joint Hospitals Committee and circulated to hospitals in August 1906.

2. That it is advisable that an annual conference should be held.

3. That a committee be appointed, not exceeding fifteen in number, to act in conjunction with the Hospitals Committee of the British Medical Association, with instructions to communicate to all hospitals the resolutions of the present conference, to arrange for the holding of a conference in 1907, and in the interval to advise and assist hospitals and kindred institutions in incorporating in their regulations the principles approved by the conference, and to deal with any other matters which may arise relating to the conference or to the objects thereof.

4. That an appeal be made to hospitals to contribute towards the expenses of carrying on the work.

